# Advanced Practitioners in Oncology: Diverse Experiences, Shared Challenges

**DOI:** 10.6004/jadpro.2014.5.2.7

**Published:** 2014-03-01

**Authors:** Susan Reckling

**Affiliations:** Susan Reckling has been a freelance medical writer and editor for more than 20 years, most of them specializing in oncology. Prior to that, she served as executive editor for two monthly medical journals.

## Report from JADPRO Live

The professional paths of advanced practitioners (APs) in oncology are as varied as the locations in which they work. At the first annual JADPRO Live educational symposium held this past January in St. Petersburg, Florida, a distinguished panel was assembled to discuss the profile of today’s APs from the unique perspectives of a nurse practitioner (NP) at a large academic cancer center, a clinical nurse specialist (CNS) and scientist, an NP at a rural cancer institute, a clinical pharmacist at a comprehensive cancer center, and a physician assistant (PA) at the oldest private cancer center in the country. The panel was led by moderator Laura Zitella, MS, RN, ACNP-BC, AOCN®.

The assortment of challenges facing APs requires clear thinking, assistance from those who have already blazed a path, and confidence to discover workable solutions. The panel focused on issues such as fostering the collaborative practice model in oncology, balancing academic education and on-the-job clinical training, establishing advanced practice groups as a forum for the voice of the AP to be heard, and serving as mentors to younger practitioners and/or students.

## Different Roads All Lead to Collaborative Models of Practice

Although the professional experiences of the panel members were diverse, many of them shared the collaborative nature of their current roles. To begin, Jeannine M. Brant, PhD, APRN, AOCN®, was a pain consultant in her first advanced practitioner role, developing a pain service with a group of NPs who managed pain in patients admitted to the hospital. In another role, Dr. Brant was involved in the development of a supportive care clinic comprising an interdisciplinary team of NPs, pharmacists, PAs, social workers, dietitians, and occupational therapists. The team meets weekly to help patients struggling with uncontrolled physical and/or psychosocial symptoms. One patient said, "I will never miss my symptom management visit because it’s the time I get to talk about my symptoms and quality of life instead of my disease."

After a general practice residency and an oncology fellowship, Christopher J. Campen, PharmD, BCPS, BCOP, worked as an academic pharmacist in conjunction with an outpatient cancer treatment center. "I was the first pharmacist to work with outpatients in the clinical arenas about 5 years ago at our cancer center," he revealed. Dr. Campen predicted that the model of the future is for pharmacists to work alongside APs, physicians, and fellows in outpatient clinics and infusion areas to reduce errors and improve patient safety. In addition to doing a lot of protocol and standard development, Dr. Campen became involved in direct patient care, counseling patients with lung cancer on the adverse effects of traditional chemotherapy and targeted agents including erlotinib.

Laura Zitella, MS, RN, ACNP-BC, AOCN®, and Heather M. Hylton, MS, PA-C, shared their positive collaborative experiences working with pharmacists. "I often grab our oncology pharmacist to go to a pain consult on the inpatient unit," said Dr. Brant. Pharmacists have been among the staunchest supporters of APs in collaborative practice. This sentiment was echoed by Ms. Hylton. "I could never practice without a clinical pharmacist," she declared.

In her administrative role, Ms. Hylton has helped to build programs and integrate APs into different services at her cancer center. In terms of her clinical role in bone marrow and stem cell transplantation, there are two separate inpatient teams, with each team having four to five PAs and NPs, an attending physician, a fellow, and a clinical pharmacist. 

Over the past 15 years, Ms. Zitella has seen much change in the bone marrow transplant setting. She recognized the importance of caring for patients across the inpatient and outpatient settings. She helped develop a program where the outpatient nurse practitioner and the inpatient fellow switched positions for 2 months of the year. Over the years, this evolved into inpatient and outpatient teams that include both advanced practitioners and fellows year-round. In the hematology/oncology divisions, she was instrumental in creating a collaborative inpatient service consisting exclusively of advanced practitioners supervised by an attending physician.

Turning to her role as a community oncology nurse practitioner, Wendy H. Vogel, MSN, FNP, AOCNP®, called her current practice model a chameleon. She is in a collaborative practice with seven oncologists, seeing a schedule of patients similar to theirs. In addition to writing and publishing her own practice protocols, Ms. Vogel transformed her passion for prevention into a high-risk cancer clinic. Partnering with a genetic counselor, she offers both genetic testing and risk-reduction counseling.

## Transforming Challenges Into Opportunities for APs

The panel agreed that many of the challenges facing today’s APs may also be viewed as opportunities. Two specific areas of concern echoed by the panel and many attendees centered on the need to improve the academic and clinical training/orientation of APs and the importance of promoting the evolving role of APs in cancer centers, in community settings, and in the eyes of the public.

**Table 1:**
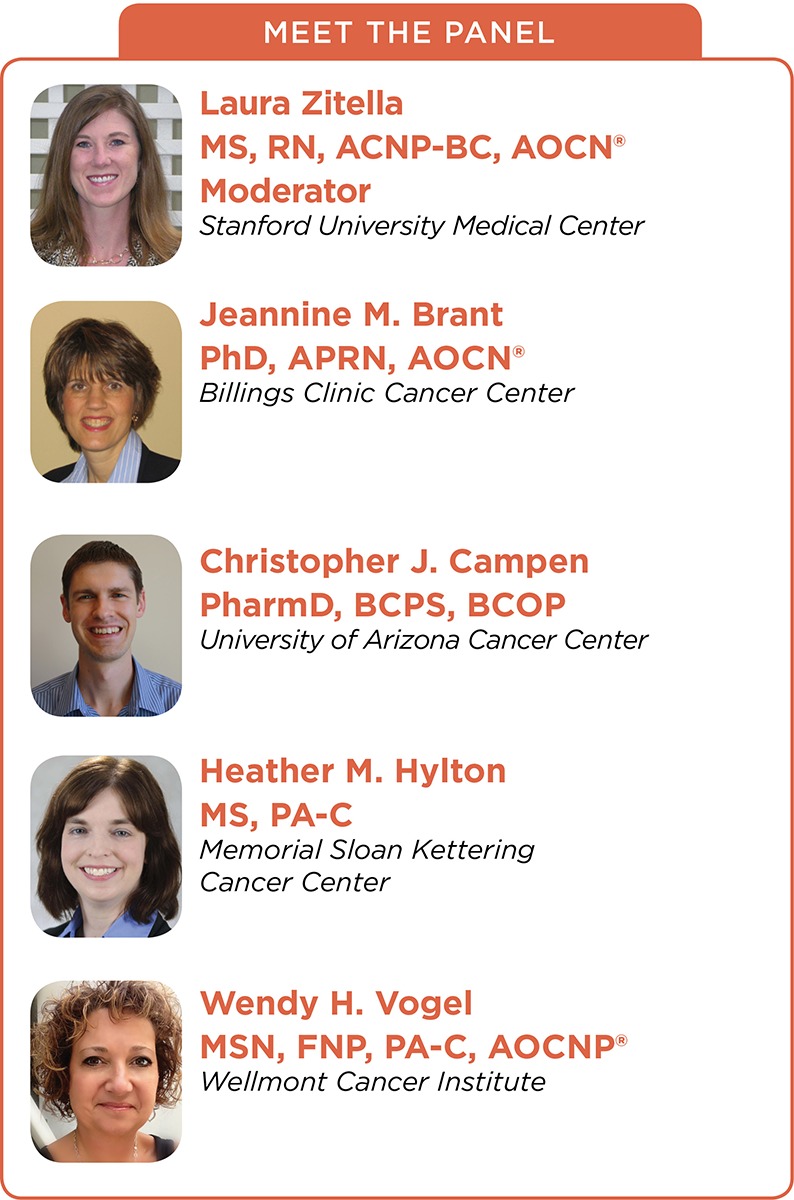


The training of APs differs from that of physicians. Ms. Zitella noted that most APs are trained as generalists, with the oncology coming as an apprenticeship and on-the-job training. Academic training for APs, including educational information from such organizations as the American Society of Clinical Oncology (ASCO) and the Oncology Nursing Society, is part of the equation, but so is adequate time for clinical training, she added.

Although the panel and many attendees considered several months to be a reasonable duration of orientation for an AP new to oncology, all admitted that in reality, the allotted time is much shorter than that. There is a struggle to obtain administrative support for proper orientation and assignment of a mentor. A strong mentoring relationship between a supervising physician and an AP is a critical part of clinical training.

The panel acknowledged the ongoing need for more hands-on training programs for APs, postgraduate programs, and resources geared for those in the community setting. Ms. Hylton mentioned ASCO’s Curricula for Advanced Practice Providers, which offers courses on topics such as basic symptom management, communication with patients, and chemotherapy administration and pharmacology. Clerkships for later-level students were also noted as a possibility, as was the practice of taking on students interested in health care. "Working with students is the best recruitment tool; I’ve hired many of my former students to be part of our team," stated Ms. Zitella.

Furthermore, much can be accomplished regarding advocacy efforts on behalf of APs. For instance, Ms. Zitella shared her experiences in creating an advanced practice provider council at Stanford. Caught between nursing and medicine and often reporting to nonclinical managers, APs found their own forum to effect change through this group, she added. Several recommendations were made by the panel to APs looking to implement changes in their practices and to serve as advocates for health policy: Have long-term goals but start with small victories, be patient and line up all of your champions, promote your passion in writing and speaking, and get involved on the local political level.

## Final Thought

In closing, the panel flagged one particular area in which collaborative practice may improve patient outcomes: adherence to oral oncolytic agents. Drs. Brant and Campen noted that there are stellar models focusing on oral adherence for multidisciplinary teams, and one of the best is a system that runs through a clinical pharmacist and an advanced practitioner. Through such collaborative efforts, patient questions can be answered, adherence and refills can be tracked either by phone or text, the need for changes in dose can be assessed, and side effects can be identified and managed through follow-up. Ms. Vogel agreed that an oral clinic would be a major benefit to both patients and providers. "These patients often do not get the education or attention that patients receiving IV chemotherapy receive," she concluded.

